# Trypsin cycling in poultry is associated with metabolic regulation

**DOI:** 10.3389/fphys.2023.1226546

**Published:** 2023-11-22

**Authors:** Vladimir G. Vertiprakhov, Vladimir I. Trukhachev, Natalya V. Ovchinnikova

**Affiliations:** ^1^ Timiryazev Russian State Agrarian University—Moscow Agrarian Academy, Moscow, Russia; ^2^ Physiology of Motivation Laboratory, Anokhin Research Institute of Normal Physiology, Moscow, Russia

**Keywords:** trypsin, chickens, duodenum, blood enzymes, enzyme cycle in poultry

## Introduction

Trypsin is one of the serine proteases and hydrolyzes proteins and peptides to amino acids. However, according to recent studies (Luo et al., 2005), the function of trypsin goes beyond enzymatic: when entering the bloodstream and with insufficient inhibitors trypsin can “trigger” the reaction cascade of coagulation, fibrinolytic and kallikrein-kinin system, also trypsin is an activator of PAR-receptors (protease-activated receptors). For a long time, it was believed that trypsin is normally synthesized only in the pancreas. A study by Koshikawa et al. (1998) showed trypsin expression in human and mouse nonpancreatic tissues. The trypsin gene was found to be expressed at high levels in the pancreas, spleen, and to a large extent in the small intestine. However, *in situ* hybridization and immunohistochemistry showed that trypsin is widely expressed in epithelial cells of the skin, esophagus, stomach, small intestine, lung, kidney, liver, and extrahepatic bile ducts, as well as in the spleen and neurons. In the spleen, trypsin activity was detected in macrophages, monocytes, and lymphocytes in the white pulp, and in the brain in nerve cells of the hippocampus and cerebral cortex. For the first time we determined trypsin activity in cow milk and found a significant increase in the enzyme activity during inflammation in the mammary gland (Vertiprakhov et al., 2022). Literature data allow us to conclude that trypsin is widespread due to in organs and tissues of the animal body, but its integrating and regulatory function has not been fully studied.

## Trypsin—a marker in determining the health of the pancreas and the body as a whole

Generalization of the results of works devoted to the study of trypsin to conclude that its determination in blood serum by radioimmune analysis (RIA) is an optimal marker for revealing the state of the pancreas (Geller and Pashko, 1990). Changes of trypsin parameters in blood serum in norm and in pathology of pancreas, mucous membranes of stomach and duodenum were studied well enough. Few studies have been devoted to changes of serum trypsin parameters in diseases of other organs and systems of the organism, except for gastrointestinal tract, including infectious pathology. According to the literature, trypsin, which belongs to proteases, is involved in the process of hemostasis and acceleration of lipid peroxidation processes. It is concluded that determination of trypsin parameters in blood serum by radioimmune analysis is an informative test of the state of not only human pancreas but also of the organism as a whole. Biochemical method for determination of trypsin activity in biological media using BAPNA substrate has found wide application in clinical diagnostics and scientific research. The stimulating effect of feeding on the activity of digestive enzymes and the content of the universal cell mediator nitric oxide in the blood plasma of Leghorn breed cockerels was established (Fisinin et al., 2018a). It was shown that the increase in trypsin activity in blood observed after feeding was In review 2 accompanied by a several-fold increase in the concentration of nitric oxide donors as judged by the Fe(NO)n content in blood. The decrease in trypsin activity observed 3 h after feeding was accompanied by a decrease in Fe(NO)n content. The increase in trypsin activity thus closely correlates with the content of deposited NO in the blood, which is evidence for the participation of trypsin in metabolic regulation processes in animals and humans along with the parasympathetic nervous system.

## Trypsin turnover in the poultry organism

The organs of the digestive system (pancreas, intestinal mucosa) produce enzymes: amylase, lipase, trypsin, alkaline phosphatase (ALP), etc. Normally, their activity in blood serum is low and constant. However, under pathology, when any of the usual ways of their excretion is blocked, the activity of these enzymes in blood serum increases significantly (Yarets, 2016). Studies carried out on healthy poultry provided data on the amount of the enzyme mediated through its activity in different biological media (Table 1).

**TABLE 1:** Trypsin activity and content of total calcium and phosphorus in the daily portion of biological fluids in chickens.

Biological environment	In the daily volume of biomaterial from chickens		
	Trypsin activity, U/L	Calcium content, g	Phosphorus content, g
duodenum	125.7	2.9	0.4
blood	22.500	0.200	0.001
feces	0.65	1.6	0.3
urine	0.24	0.02	0.002

In this table, we provide purely mathematical calculations of trypsin activity in different biomaterials from chickens to understand how much the activity of the enzyme changes in the respective biological environment. Considering that different biomaterials contain trypsin inhibitors, accounting for them in this paper would make it difficult to determine the dynamics of trypsin movement in poultry. Although it is known (Vertiprakhov et al., 2017) that trypsin inhibitors are present in all biological media, regulating enzyme activity and preserving the integrity of living tissues, inhibitor activity stabilizes with age (after 7 days of age). The pancreas of chickens secretes 45 mL of pancreatic juice with trypsin activity of 2,794 U/L (Vertiprakhov and Grozina, 2018). If calculated at 45 mL (pancreatic juice per day), it turns out that broilers secrete 125.7 U/L per day, which far exceeds the requirement for trypsin to break down 10 g of feed protein. According to Batoev (2001), Batoev (2016) the daily amount of juice in chickens can break down 27 g of casein in 1 min, which is the protein requirement for 2 days. It is known that part of the enzyme enters from the intestine into the blood and is sent to the pancreas to form a new secretion, which will include two pools of enzymes: newly secreted and those returned from the intestine, tissue fluid, etc. (Korotko et al., 1986; Rothman et al., 2002). Trypsin activity in chicken blood averages 150 U/L (Fisinin et al., 2018b). The amount of blood in a 1.5 kg hen averages 150 mL, so the average trypsin activity in the blood volume will be 22.5 U/L. It was found that in blood as well as in intestine trypsin activity changes in the postprandial phase of digestion, as well as with the age of the bird (Fisinin et al., 2018a; Vertiprakhov and Ovchinnikova, 2022).

Table 1 shows the content of calcium, which can be considered as a trypsin inhibitor (Kryukov et al., 2021), and phosphorus as a macronutrient that stimulates protein metabolism and protease activity. These findings are in agreement with the results of a study by Batoev, who found a positive correlation between digestive enzymes and phosphorus and a negative correlation between trypsin and calcium (Batoev, 2001; Batoev, 2016).

Chickens excrete on average 13.6 g of absolutely dry feces with trypsin activity equal to 48.2 units/liter per day, i.e., one hen excretes trypsin activity equal to 0.65 U/L. Consequently, if we take trypsin activity in the duodenum as the maximum amount, 0.5% of trypsin activity is excreted with the litter. In the literature, there are data that trypsin activity is significantly reduced (up to 15%) when passing the intestine (Vertiprakhov et al., 2020). This may be due to a variety of reasons. A significant decrease in the activity of proteases as they move through the digestive canal can be explained by the return of enzymes into the blood according to the pancreatic enzyme recreation hypothesis (Rothman et al., 2002; Korotko, 2011) or their destruction by intestinal microflora.

The amount of urine per day in chickens is on average 60 mL, trypsin activity in it is on average 4 U/L (Vertiprakhov et al., 2023). A calculation per 60 mL yields 0.24 U/L.

Enzyme activity is the rate of an enzymatic reaction divided by the concentration of the enzyme. Enzymes can be in different functional states, so the parameters “enzyme activity” and “enzyme amount” may not coincide. Enzyme activity characterizes the efficiency of an enzyme rather than the quantity (Batoev, 2016). Using data from the literature, we tried to determine the number of enzymes that are excreted with various biological fluids in the body per day. If we consider that 60 g of protein is excreted in the human digestive tract per day, then we can determine the amount of protein excreted with pancreatic juice per day, based on the role of the pancreas. It is equal to 7 g, because in a day a person excretes on average 700 mL of pancreatic juice with protein content of 1.0% (Shlygin, 1974). It is believed that pancreatic juice is more than 90% water and contains protein about 190–300 mg/100 mL of juice, which as a percentage is 0.2%–0.3% (Sablin et al., 2002).

The amount of protein in the pancreatic juice of chickens is 23 ± 2.2 g/L, or 2.3% (Smolin, 2008; Grozina, 2019). Taking into account that daily pancreatic juice excretion in a chicken is 28 mL, the protein content is no more than 0.64 g. Part of the plasma protein is accounted for by enzymes. Considering that the amount of blood in a chicken averages 150 mL, the number of enzymes is 0.15 g or 0.1% of the total amount of protein in the blood plasma of the bird.

The intestinal microflora take part in the inactivation of the pancreatic juice enzymes trypsin and amylase (Batoev, 2001). Nevertheless, trypsin activity in the feces of ileal birds is 48 units/L. In the feces of chickens, the amount of total protein removed from the intestine per day as part of digestive enzymes is 0.003 g and in urine 0.005 g per day (Vertiprakhov, 2022).

Thus, most of the pancreatic enzymes, including trypsin, remain in the bird and are returned through the blood to the pancreas. In the blood they perform a function of which the following says the following: “Proteinases must now be regarded as important hormonelike substances signaling to cells and tissues of many changes in norm and pathology. With the recognition of the signaling role of proteinases, their biology and physiology will evolve, and exciting developments await us in these areas.” (Ramachandran and Hollenberg, 2008).

## Confirmation of the hypothesis based on the calculated data of pancreatic enzyme synthesis *de novo*

The review (Mozheiko, 2017) distinguishes 2 types of exocrine pancreatic secretion: the interdigestive (basal) type, which makes up about 10% of the total secretory activity of the organ, and the postprandial (stimulated by food intake) type, which makes up 80%–90%. These types differ significantly in the amount and enzymatic activity of pancreatic juice (Keller and Layer, 2005; Chandra and Liddle, 2015). Outside of digestion, a small amount of pancreatic juice containing amylo-, proteo- and lipolytic enzymes is secreted into the cavity of the duodenum. Exocrine pancreatocytes are in the phase of reduced physiological activity. It is believed that synthesis and repair processes in the secretory cells are continuous, and extrusion is periodic, based on endogenous rhythms. After a meal, there is a sharp intensification of secretion, which is realized in three consecutive phases. Secretory activity in the first (cerebral) phase is stimulated by conditionally and unconditioned reflex way. The amount of pancreatic juice in the first phase is 10%–15% of the total volume of secretion during the entire digestive period. And the enzyme secretion reaches 25%. The second (gastric) phase of pancreatic secretion does not exceed 10% of its total volume and is characterized by a high concentration of enzymes in the juice. The third (intestinal) phase accounts for 75%–85% of the pancreatic juice volume with high bicarbonate content (Smirnov, 2002; Chandra and Liddle, 2015). It is traditionally believed that after a meal, the pancreas completely synthesizes a new composition of enzymes, which are secreted into the small intestine. To ensure digestion in the intestine, exocrine pancreatocytes have a high secretory protein capacity that has no equal in our body. The 12 duodenum receives 6–20 mg of pancreatic enzymes per day (Chandra and Liddle, 2015). They are synthesized at a rate of 170 enzyme molecules per minute. Autoradiographic studies combined with electron microscopy showed that labeled amino acids are determined in the granular endoplasmic network 5 min after injection, and in 20 min - in elements of the Golgi complex. Another 40 min, according to *in vitro* and *in vivo* experiments, are required for the final formation of secretory granules (Permyakov et al., 1973). Exocytosis occurs at a high rate. It takes no more than 7 s to eliminate the contents of a single pellet (Chandra and Liddle, 2015). Despite this, when comparing the number of enzymes newly synthesized in exocrine pancreatocytes with the number of enzymes excreted in pancreatic juice, a clear discrepancy was found (Rothman et al., 2002).

This suggested that Postprandial synthesis of newly synthesized enzymes is still insufficient to fully supply pancreatic juice (Rothman, 1975). To assess the ability of the exocrine part of the pancreas to fully resynthesize the required number of enzymes, several ways have been considered. For example, the rate of synthesis of digestive enzymes is compared with the rate at which they are excreted into the intestine (Rothman et al., 2002). Based on data obtained in anesthetized rats after overnight fasting, the amount of protein secreted by the pancreas is defined as The amount of protein secreted by the pancreas is 70–140 mg per 1 g of tissue daily (Miyasaka and Rothman, 1982; Ho and Rothman, 1983). In the absence of exogenous stimuli, the basal secretion rate is ∼0.25–1.0 mg per 1 g of tissue per hour (Rothman, 1975), i.e., the pancreas can produce from 6 to 24 mg of protein per day per 1 g of tissue, which is 4%–35% of the protein secreted into the intestine (Rothman et al., 2002). The authors concluded that with even the highest rate of enzyme synthesis in pancreatocytes (1 mg/g/hr) and the lowest rate of their excretion into the intestine (70 mg/g/day) for the calculations, the former rate remains three times less than the latter. According to a number of literature data, the rate of enzyme synthesis in the pancreas after administration of secretion stimulants increases by 25%–50% (Grendell and Rothman, 1983). However, this is not enough to compensate for the resulting deficit. In addition, it has been shown that during periods of active secretion, the content of enzymes in pancreatic tissue decreases significantly significantly. Thus, after 3 h of active postprandial secretion, the enzyme content decreases by half (∼35 mg/g/day) and, according to calculations, it takes about 60 h of proteosynthesis to compensate for this loss and return to baseline synthesis levels (Ho and Rothman, 1983). It has been it has also been suggested that the synthetic capacity of pancreatocytes be evaluated by the rate of synthesis of a single peptide chain and the number of ribosomes actively involved. It was found that the synthesis of a single peptide chain takes ∼3 min to synthesize one molecule of pancreatic ribonuclease or amylase (Redman et al., 1966), i.e., 20 protein molecules are synthesized per hour. These are synthesized exclusively in ribosomes associated with the endoplasmic reticulum, which in exocrine pancreatocytes occupies about 20% of the cell volume (Permyakov et al., 1973; Klimov and Fokina, 1987). Thus, 1 g tissue is capable of synthesizing ∼0.67 mg of digestive enzymes per hour, or, under other assumptions, 0.34 mg 0.34 mg/h, corresponding to 8–16 mg per 1 g of tissue per day, or about 6%–24% of the amount excreted Of the amount excreted into the intestine. If we use the highest value for synthesis rate and the lowest value for excretion in these calculations, it is four times less than is expected to equalize them (Rothman et al., 2002). Finally, to judge the ability of the pancreas to re-synthesize the entire pool of secreted enzymes, it is suggested that its energy potential be considered. The main energy expenditures during transport of necessary substances through the plasma membrane of acinar cells, as well as from small vesicles to the condensation vacuoles of the Golgi complex and during mature granule formation are compensated during oxidative phosphorylation in mitochondria (Permyakov et al., 1973). A number of authors have estimated that it takes 3.5 kJ/g of energy to form one peptide bond is required to form one peptide bond of a protein (Simon et al., 1978; Reeds et al., 1980). Energy energy capacity of the pancreas, determined experimentally by oxygen consumption, ranges from 17 to 37 J per g of tissue per day in rats. This indicates that the pancreas is a metabolically active organ. Nevertheless, to replace the 70 mg/g tissue pool of secreted enzymes in starving rats (basal secretion) per day requires 238 J per g tissue. secretion) per day requires 238 J, i.e., almost 10 times the energy capacity of the cells, which, calculated to provide only 4%–15% of the actual number of enzymes secreted into secretion. This is by the highest synthesis estimates and the lowest secretion values 7 times less than required (Rothman et al., 2002).

As a result, using different approaches to estimate pancreatic secretory capacity were obtained exactly the same results, which, according to the authors, confirms the correctness of their assumptions (Rothman et al., 2002).

## Discussion

So, trypsin in animals is secreted in the pancreas. In the intestine, it is involved in the hydrolysis of proteins to peptides and amino acids, and subsequently enters the blood. Due to the transport function of the blood, it is distributed throughout the body. Expression of the enzyme in many organs and tissues, as well as the presence of special PAR-receptors indicates its participation in the regulation of body functions. The data obtained on the circulation of trypsin in the body of chickens clearly showed that its hormone-like action is not limited to the function of the pancreas, but is aimed at the regulation of metabolism throughout the body by optimizing the content of free amino acids in different biological media.
